# A Pilot Study Using Frequent Inpatient Assessments of Suicidal Thinking to Predict Short-Term Postdischarge Suicidal Behavior

**DOI:** 10.1001/jamanetworkopen.2021.0591

**Published:** 2021-03-09

**Authors:** Shirley B. Wang, Daniel D. L. Coppersmith, Evan M. Kleiman, Kate H. Bentley, Alexander J. Millner, Rebecca Fortgang, Patrick Mair, Walter Dempsey, Jeff C. Huffman, Matthew K. Nock

**Affiliations:** 1 Department of Psychology, Harvard University, Cambridge, Massachusetts; 2 Department of Psychology, Rutgers University, New Brunswick, New Jersey; 3 Department of Psychiatry, Massachusetts General Hospital, Boston; 4 Mental Health Research, Franciscan Children’s, Brighton, Massachusetts; 5 Department of Biostatistics, University of Michigan, Ann Arbor

## Abstract

**Importance:**

The weeks following discharge from psychiatric hospitalization are the highest-risk period for suicide attempts. Real-time monitoring of suicidal thoughts via smartphone prompts may be more indicative of short-term risk than a single, cross-sectional assessment.

**Objective:**

To test whether modeling dynamic changes in real-time suicidal thoughts during psychiatric hospitalization can improve predictions of postdischarge suicide attempts vs using only baseline (ie, admission) data or using the mean level of real-time suicidal thoughts during hospitalization.

**Design, Setting, and Participants:**

In this prognostic study, 83 adults recruited from the inpatient psychiatric unit at Massachusetts General Hospital completed ecological momentary assessment surveys of suicidal thinking 4 to 6 times per day during hospitalization as well as brief follow-up surveys assessing suicide attempts at 2 and 4 weeks after discharge. Participants completed at least 3 real-time monitoring surveys. Inclusion criteria included hospitalization for suicidal thoughts and/or behaviors and English fluency. Data were collected from January 2016 to December 2018 and analyzed from January to December 2020.

**Main Outcomes and Measures:**

The primary outcome was suicide attempt in the month after discharge.

**Results:**

Of 83 participants (mean [SD] age, 38.4 [13.6] years; 43 [51.8%] male participants; 69 [83.1%] White individuals), 9 (10.8%) made a suicide attempt in the month after discharge. Mean cross-validated AUC for elastic net models revealed predictive accuracy was fair for the model using baseline data (area under the curve [AUC], 0.71; first to third quartile, 0.55-0.88), good for the model using the mean level of real-time suicidal thoughts during hospitalization (AUC, 0.81; first to third quartile, 0.67-0.91), and best for the model using dynamic changes in real-time suicidal thoughts during hospitalization (AUC, 0.89; first to third quartile, 0.81-0.97); this pattern of results held for other classification metrics (eg, accuracy, positive predictive value, Brier score) and when using different cross-validation procedures. Features assessing rapid fluctuations in suicidal thinking emerged as the strongest predictors of posthospital suicide attempts. A final set of models incorporating percentage missingness further improved both the mean (mean AUC, 0.93; first to third quartile, 0.90-1.00) and dynamic feature (mean AUC, 0.93; first to third quartile, 0.88-1.00) models.

**Conclusions and Relevance:**

In this study, collecting real-time data about suicidal thinking during the course of hospitalization significantly improved short-term prediction of posthospitalization suicide attempts. Models including dynamic changes in suicidal thinking over time yielded the best prediction; features that captured rapid changes in suicidal thoughts were particularly strong predictors. Survey noncompletion also emerged as an important predictor of posthospitalization suicide attempts.

## Key Points

**Question:**

Can prediction of suicide attempts after psychiatric hospitalization be improved using frequent assessments of the level and variability of an individual’s suicidal thoughts?

**Findings:**

In this prognostic study of 83 adult psychiatric inpatients, prediction of posthospital suicide attempts was fair when using only baseline data, improved in a model using mean level of suicidal thinking during hospitalization, and improved further in a model including dynamic features of suicidal thoughts.

**Meaning:**

These findings suggest that data on real-time, dynamic changes in suicidal thoughts could improve prediction of suicide attempts during the high-risk period following psychiatric hospitalization.

## Teaser

This prognostic study tests whether modeling dynamic changes in real-time suicidal thoughts during psychiatric hospitalization can improve predictions of postdischarge suicide attempts vs using only baseline data or using the mean level of real-time suicidal thoughts during hospitalization.

## Introduction

Suicide is a leading cause of death, claiming more than 800 000 lives each year globally and more than 45 000 in the United States.^1,2^ The highest-risk period is immediately following discharge from psychiatric care,^3,4^ with the rate of suicide attempts in the first 3 months following discharge being higher than the next 5 years combined. This risk is particularly heightened among patients admitted with suicidal thoughts or behaviors, with the rate of suicide for these patients being nearly 200 times the global suicide rate.^5^ One factor limiting our ability to predict posthospitalization suicide attempts is that few studies have observed individuals during these short-term, high-risk time windows. A 2017 meta-analysis of the past 50 years of suicide research^6^ revealed only 1% of predictive analyses used time windows of less than 30 days, usually with just 1 assessment in that window. These studies fail to capture the short-term dynamic nature of suicidal thinking and cannot provide data on short-term risk of suicide attempts, particularly those following hospitalization.

Recent advances in real-time monitoring technology provide opportunities to address these gaps via finer-grained ecological momentary assessment (EMA) of changes in suicide risk over shorter timeframes.^7,8,9,10^ These methods have revealed that suicidal thoughts are characterized by large within-person fluctuations.^7,9^ Moreover, prior retrospective work suggests that suicide attempts are preceded by large, rapid changes in suicidal thinking.^11,12^ However, no studies have tested whether real-time measurement of changes in suicidal thinking can prospectively predict imminent suicide attempts, which could inform resource allocation for patients most at risk, in a setting where care is already accessible.^13^ Currently, clinical decision-making regarding hospital discharges is informed by patients’ self-report of suicidal thinking during specific clinical assessments (eg, therapy appointments). Brief standardized assessments of suicidal thinking administered continuously throughout hospitalization may aid clinical decision-making by providing novel information about a patient’s likelihood of suicide attempt following discharge.

To advance the understanding of suicide in this high-risk period, we tested several different approaches to predicting posthospital suicide attempts using the following approaches: (1) baseline data at inpatient admission, (2) mean level of suicidal thinking during hospitalization, and (3) dynamic patterns of suicidal thinking during hospitalization. We hypothesized that computing and modeling dynamic fluctuations in suicidal thinking would yield improved predictive accuracy beyond both traditional static approaches of assessing suicidal thoughts (baseline model) and modeling only the mean of real-time suicidal thoughts during hospitalization (mean model). In addition, given evidence suggesting that missing data about suicidal thoughts may be an important predictor of suicide attempts,^14^ we also built a second series of models as an exploratory aim to examine whether using missingness as a predictor would further improve accuracy of the mean and dynamic feature models.

## Methods

In this prognostic study, we aimed to develop a model predicting posthospital suicide attempts using EMA data. All study procedures were approved by the Massachusetts General Hospital institutional review board, and all participants provided written informed consent. This study followed the Transparent Reporting of a Multivariable Prediction Model for Individual Prognosis or Diagnosis (TRIPOD) reporting guideline.^15^

### Participants

Participants were adults hospitalized at the inpatient psychiatric unit at Massachusetts General Hospital. Of 104 participants enrolled, 83 participants (79.8%) completed at least 3 EMA surveys and were included in current analyses.

### Procedure

Inclusion criteria for the study were admission to the psychiatric inpatient unit due to suicidal thoughts and/or risk (determined by patient self-report and clinician judgment) and English fluency. Exclusion criteria included any factors impairing participant ability to provide informed consent or participate in study procedures (eg, cognitive impairments). Having a compatible smartphone was not an inclusion criterion; we provided a loaner phone to participants if needed. Recruitment for this study occurred in 2 waves, with 45 participants recruited in wave 1 (January 2016 through January 2017) and 59 participants in wave 2 (May 2017 through December 2018). Both waves used identical study protocols (with the exception of EMA survey software and frequency of assessments); we pooled data from both waves in the current study.

Participants who met inclusion criteria and provided written consent to be in the study completed a baseline assessment during hospital admission. Participants then completed an EMA protocol for the duration of their hospital stay (mean [SD], 6.9 [5.4] days; range, 2-46 days) with survey prompts sent to participants’ smartphones 4 to 6 times per day during waking hours. Participants were compensated $10 per day. Finally, participants completed follow-up assessments at 2 weeks and 4 weeks following discharge.

### Measures

#### Baseline Assessment of Self-injurious Thoughts and Behaviors

Participants completed the self-report version of the Self-injurious Thoughts and Behaviors Interview (SITBI).^16^ We used the following characteristics for suicidal thoughts, plans, attempts, and nonsuicidal self-injury: lifetime presence, past-year frequency, past-month frequency, past-week frequency, and self-reported likelihood of future SITB engagement.

#### EMA of Suicidal Thoughts

In wave 1, we used moviesensXS to send participants 4 semirandom prompts per day. In wave 2, we used Beiwe to send participants 6 semirandom prompts per day to obtain greater temporal granularity. Both waves used 3 items^17^ to assess momentary suicidal thoughts on a scale from 0 (none) to 9 (very much). These were (1) desire to die by suicide (“How intense is your desire to kill yourself right now?”), (2) intent to die by suicide (“How strong is your intention to kill yourself right now?”), and (3) ability to resist the urge to die by suicide (“How strong is your ability to resist the urge to kill yourself right now?”).

#### Follow-up Assessment

We assessed presence of a suicide attempt in the month after hospitalization with 2 methods, which is common in longitudinal suicide research.^18,19,20^ First, we used follow-up surveys at 2 and 4 weeks after discharge and categorized participants as having made a suicide attempt if they (1) reported hospital admission and responded “tried to kill yourself” to the question “What was the reason for your admission overnight to a hospital for mental health care?” or (2) responded yes to the question, “In the past 2 [4] weeks (that is, since you left the unit), did you make a suicide attempt (that is, purposefully hurt yourself with at least some intent to die)?” Second, we examined the electronic medical records of each participant to determine whether they returned to the hospital due to a suicide attempt in the month following discharge.

### Statistical Analysis

#### Modeling Approach

Analyses were performed in R version 3.6.1 (R Project for Statistical Computing)^21^ via tsfeaturex,^22^ caret,^23^ and glmnet^24^ packages. We built 3 elastic net models (baseline model, mean model, dynamic feature model) predicting posthospital suicide attempts, using the default threshold of 0.5 in glmnet. We used the elastic net algorithm, given substantial evidence that penalization improves prediction accuracy,^25,26,27^ alongside its well-established accuracy and robustness^28,29^ and ability to maintain clinical interpretability compared with less transparent machine learning algorithms (eg, random forests, neural networks).

For the baseline model, we used the 20 baseline SITBI characteristics as predictors; 2.16% of data was missing and imputed with K–nearest neighbor imputation. The mean model included the mean of each EMA suicide item (ie, desire to kill self, intent to kill self, and ability to resist the urge to kill self) as predictors. For the dynamic feature model, we extracted 24 time-series features for each EMA suicide item for each participant as predictors. These features characterize magnitude, speed, and probability of changes in suicidal thoughts over time, including the minimum, maximum, mean, standard deviation, percentage unique values (ie, number of unique response values divided by total number of responses), mean square successive difference (ie, variance of differences from an observation to the next), probability of acute change (ie, number of acute changes divided by total number of changes), and maximum change (ie, from an observation to the next). Acute changes were defined as any successive change from an EMA prompt to the next in the 90th percentile or greater of the total distribution of change for each participant (ie, a large shift from 1 observation to the next).^30^

Following recommendations by Kuhn and Johnson,^31^ we used 5-fold cross-validation with 3 repetitions to select the optimal λ (shrinkage) and α (mixing) parameters for each elastic net model. This also allowed us to obtain a cross-validation estimate of model performance. Whereas splitting a data set into a single training and testing set can have limited ability to accurately characterize uncertainty in results, particularly for smaller samples, repeating the training and testing process can provide more reasonable estimates of model accuracy in predicting outcomes in new data sets. Finally, we evaluated variable importance for all models using the varImp() function in caret and extracted coefficients for the best model. Because no null hypothesis statistical testing was preformed, no prespecified level of statistical significance was set.

#### Model Performance

Our primary model performance metric was the mean cross-validation estimate of area under the curve (AUC), a discrimination metric that ranges from 0.5 (chance) to 1.0 (perfect). We also evaluated several other metrics, including the mean cross-validated area under the precision-recall curve (AUPRC), accuracy, positive predictive value (PPV), sensitivity, specificity, Brier score, and Cohen κ. Higher scores for each of these metrics indicate better model performance, with the exception of Brier score, for which lower scores indicate better model performance.

#### Secondary Models

Primary models with EMA data were built using all available data with no imputation of missing EMA data. As a secondary aim, we fit elastic net models using missingness (calculated as percentage compliance) as an additional predictor for the mean and dynamic feature model. To evaluate model robustness, we also fit our 3 primary models (baseline, mean, dynamic feature model) using 2 different cross-validation procedures, ie, 10-fold repeated 3 times and leave-one-out-cross-validation.

## Results

### Demographic and Clinical Characteristics

Participants’ demographic characteristics, diagnoses, and history of suicidal thoughts and behaviors on hospital admission are presented in the Table. In the hospital, participants completed a total of 1374 EMA surveys, with a median of 18.50 responses per participant (range, 3-120). Mean compliance rate was 52.2% (SD, 18.7%; range, 7.8%-96.2%). eFigure 1 in the Supplement presents individual time series plots of EMA responses during hospitalization. In the month following discharge, 9 participants (10.8%) made a suicide attempt; 6 (66.7%) were self-reported on a follow-up survey (with 65 participants [78.3%] completing a follow-up survey), and 3 (33.3%) were identified through electronic medical record review.

**Table.** Demographic Characteristics, Diagnoses, and Baseline Clinical Characteristics for 83 Participants^a^

Characteristic	No. (%)
Age, mean (SD), y	38.43 (13.64)
Race	
White	69 (83.1)
Black	4 (4.8)
Asian	4 (4.8)
Other^b^	5 (6.0)
Ethnicity	
Hispanic or Latino/a	7 (8.4)
Gender	
Men	43 (51.8)
Women	35 (42.2)
Transgender	3 (3.6)
Other	2 (2.4)
Any mood disorder	79 (95.2)
Any anxiety disorder	30 (36.1)
Any personality disorder	33 (39.8)
Any substance use disorder	30 (36.1)
Any eating disorder	2 (2.4)
Any psychotic disorder	3 (3.6)
ADHD	3 (3.6)
Lifetime presence of suicidal thoughts, mean (SD)	
No. (%)	81 (98.8)
Past-year frequency	43.38 (140.19)
Past-month frequency	11.58 (45.99)
Past-week frequency	3.47 (4.37)
Likelihood^c^	2.76 (1.22)
Lifetime presence of suicide plan, mean (SD)	
No. (%)	64 (78.0)
Past-year frequency	4.21 (8.24)
Past-month frequency	1.68 (2.74)
Past-week frequency	1.01 (1.76)
Likelihood^c^	2.26 (1.35)
Lifetime presence of suicide attempt, mean (SD)	
No. (%)	62 (75.6)
Past-year frequency	1.31 (2.51)
Past-month frequency	0.64 (0.87)
Past-week frequency	0.38 (0.60)
Likelihood^c^	2.04 (1.35)
Lifetime presence of NSSI, mean (SD)	
No. (%)	32 (39.0)
Past-year frequency	2.86 (7.73)
Past-month frequency	0.89 (3.05)
Past-week frequency	0.21 (0.56)
Likelihood^c^	1.58 (1.57)

Abbreviations: ADHD, attention-deficit/hyperactivity disorder; NSSI, nonsuicidal self-injury.

^a^ The baseline characteristics of suicidal thoughts and behaviors includes data from 82 participants, as 1 participants did not complete this baseline survey.

^b^ Write-in responses for other races included Native American, Puerto Rican, Dominican, and Honduran.

^c^ Likelihood indicates self-reported likelihood of future suicidal thoughts, plans, attempts, and NSSI, rated from 0 (not at all) to 4 (very likely).

### Model Performance

**Figure 1.**
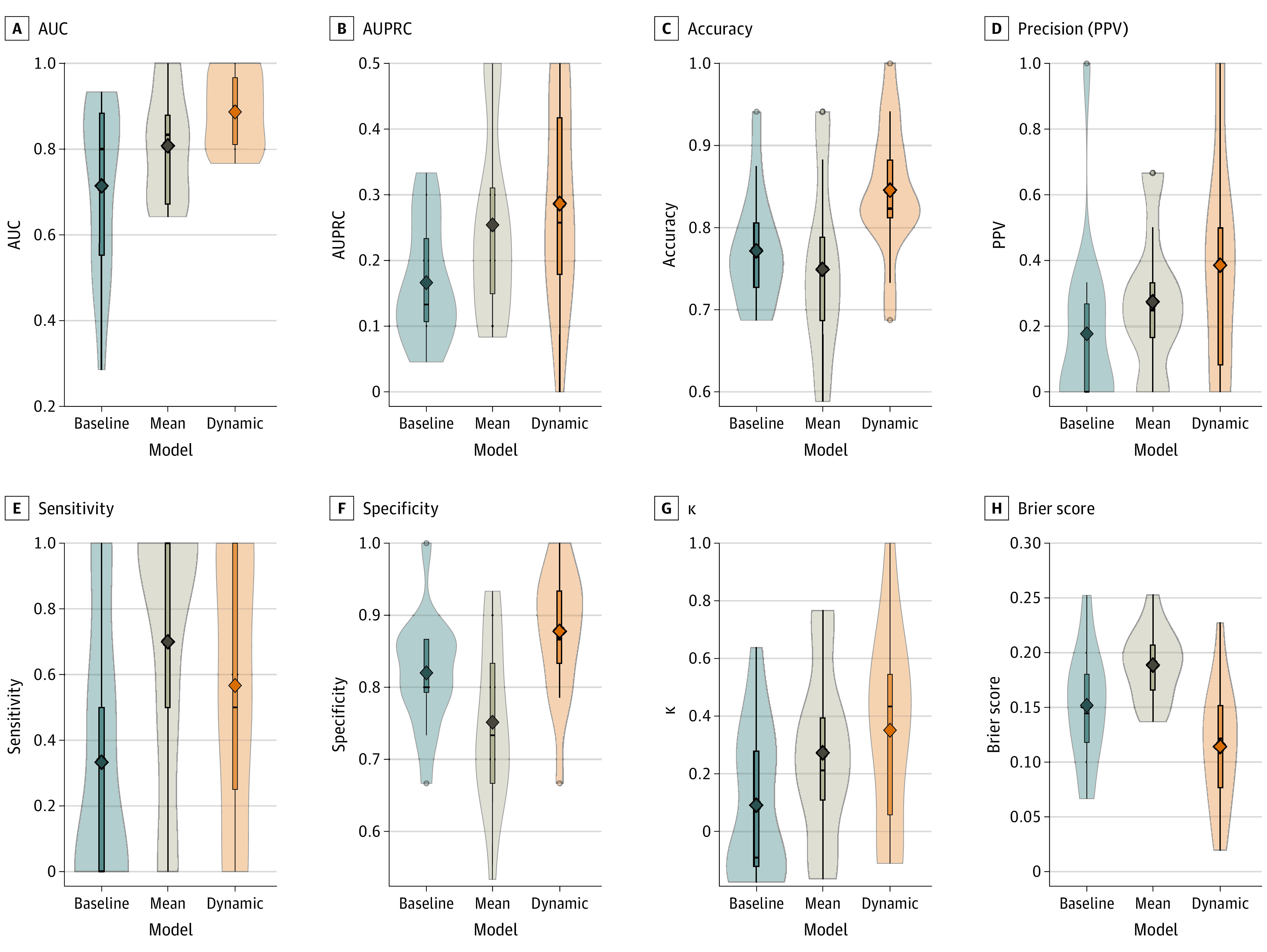
Suicide Attempt Prediction Model Metrics Each panel presents descriptive visualizations (violin plots) of the distribution of cross-validated model performance metrics. The darker shaded boxes within each violin plot are box plots, with the lower and upper hinges representing the first and third quartiles, respectively, and the whiskers extending to the most extreme data points within 1.5 times of the interquartile range. Data points beyond the whiskers are plotted as separate points. AUC indicates area under the curve; AUPRC, area under the precision-recall curve; and PPV, positive predictive value.

Model performance metrics for the baseline, mean, and dynamic models are presented in Figure 1. The baseline model provided fair AUC (mean cross-validated AUC, 0.71; interquartile range [IQR], 0.55 to 0.88), with poor AUPRC (mean, 0.17; IQR, 0.11 to 0.23), poor accuracy (mean, 0.77; IQR, 0.73 to 0.81), poor PPV (mean, 0.18; IQR, 0.00 to 0.27), poor sensitivity (mean, 0.33; IQR, 0.00 to 0.50), fair specificity (mean, 0.82; IQR, 0.79 to 0.87), fair Brier score (mean, 0.14; IQR, 0.14 to 0.18), and poor κ (mean, 0.09; IQR, −0.12 to 0.28). Of note, 1 participant who made a posthospitalization suicide attempt did not complete the baseline survey. Thus, 82 participants were included in this model. The mean model provided better performance than the baseline model, with good AUC (mean, 0.81, IQR, 0.67-0.91), but poor AUPRC (mean, 0.25; IQR, 0.15-0.31), fair accuracy (mean, 0.75; IQR, 0.69-0.79), poor PPV (mean, 0.28; IQR, 0.17-0.33), fair sensitivity (mean, 0.70; IQR, 0.50-1.00), fair specificity (mean, 0.75; IQR, 0.67-0.83), good Brier score (mean, 0.17; IQR, 0.14-0.20), and fair κ (mean, 0.27; IQR, 0.11-0.39). The dynamic feature model provided a large improvement over the baseline model and further improved on the mean model, with good AUC (mean, 0.89; IQR, 0.81-0.97]), poor AUPRC (mean, 0.29; IQR, 0.18-0.42), good accuracy (mean, 0.85; IQR, 0.81-0.88), fair PPV (mean, 0.39; IQR, 0.08-0.50), poor sensitivity (mean, 0.57; IQR, 0.25-1.00), good specificity (mean, 0.88; IQR, 0.83-0.93), good Brier score (mean, 0.11; IQR, 0.08-0.15), and fair κ (mean, 0.35; IQR, 0.06-0.54).

### Predictor Importance

**Figure 2.**
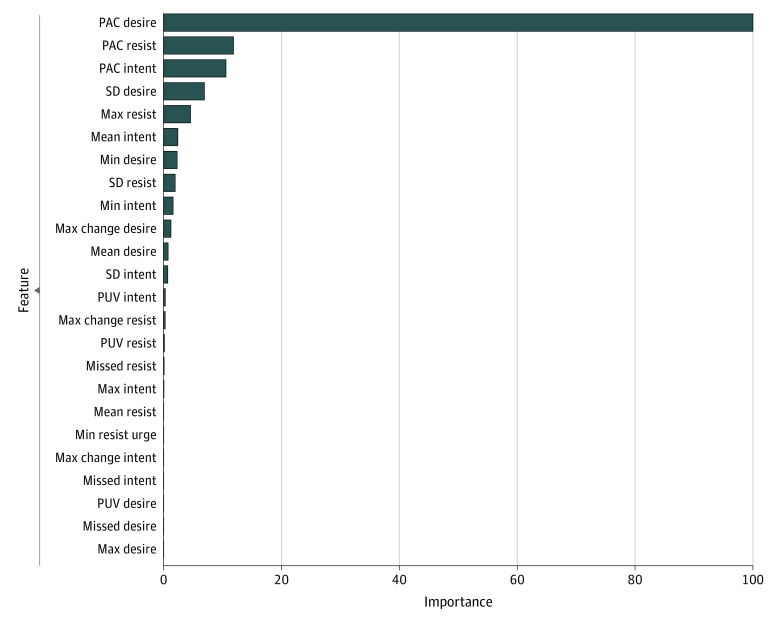
Dynamic Feature Model Variable Importance Importance scores are coefficients for the final model scaled from 0 to 100, with 0 indicating least important and 100 indicating most important. Max indicates maximum; min, minimum; PAC, probability of acute change; PUV, percentage unique values.

Variable importance metrics (ie, coefficients for the final model scaled from 0 to 100, with 0 indicating least important and 100 indicating most important) for the dynamic model are shown in Figure 2; model coefficients for the best model appear in the eTable in the Supplement. The most important EMA time-series features for predicting posthospitalization suicide attempts included probability of acute change in the desire, intention, and ability to resist the urge to kill oneself.

### Secondary Models

When using different cross-validation procedures to evaluate model robustness, results largely remained unchanged. For both 10-fold cross-validation and leave-one-out-cross-validation, the baseline model provided poor or fair performance, the mean model provided better performance, and the dynamic feature model performed the best (eFigure 2 and eFigure 3 in the Supplement) across nearly all classification metrics.

**Figure 3.**
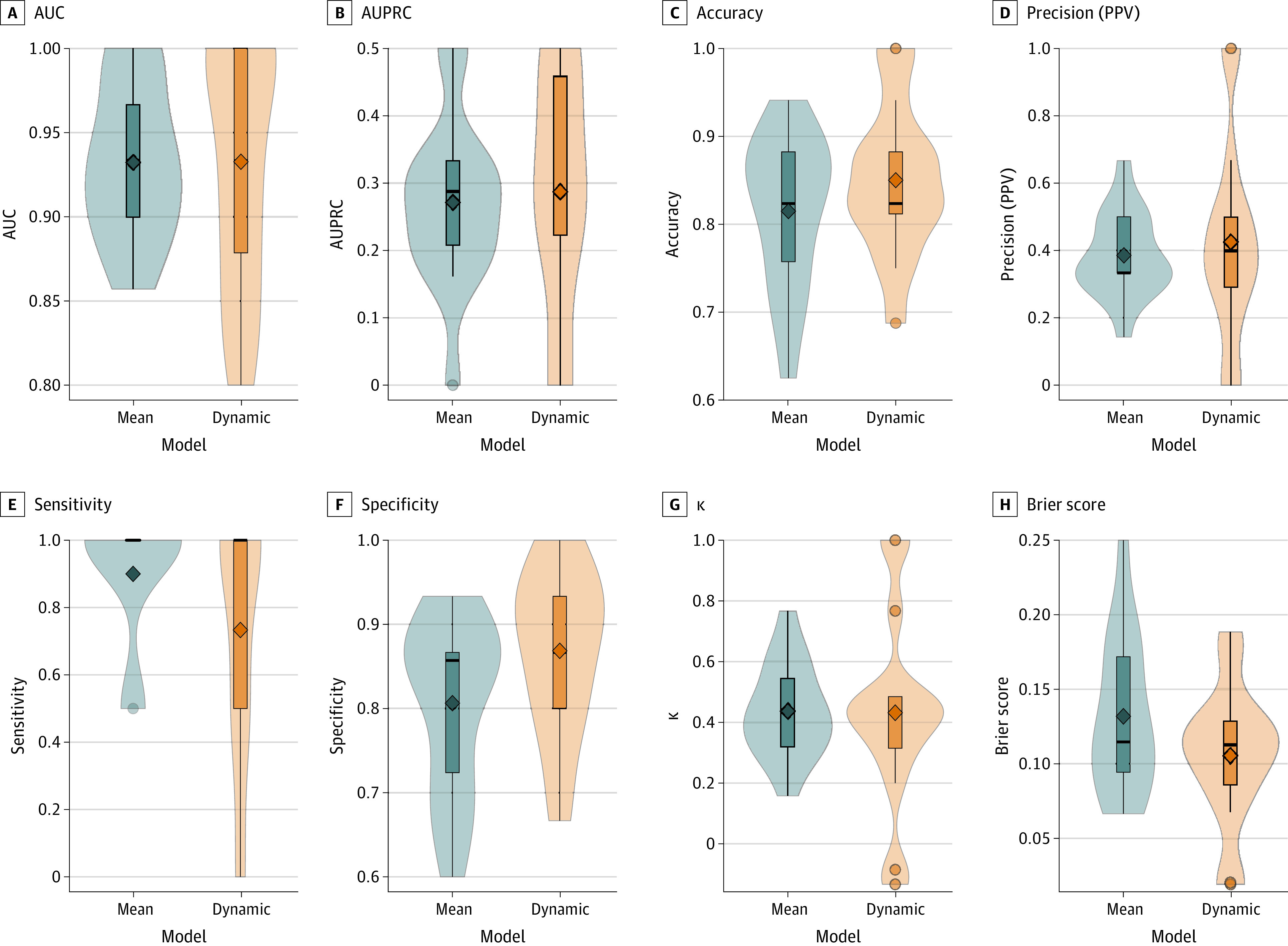
Suicide Attempt Prediction Model Metrics, With Missingness Added Each panel presents descriptive visualizations (violin plots) of the distribution of cross-validated model performance metrics. The darker shaded boxes within each violin plot are box plots, with the lower and upper hinges representing the first and third quartiles, respectively, and the whiskers extending to the most extreme data points within 1.5 times of the interquartile range. Data points beyond the whiskers are plotted as separate points. AUC indicates area under the curve; AUPRC, area under the precision-recall curve; and PPV, positive predictive value.

Adding EMA missingness as a predictor improved mean AUC for both the mean (mean AUC, 0.93; first to third quartile, 0.90-1.00) and dynamic feature (mean AUC, 0.93; first to third quartile, 0.88-1.00) models (Figure 3). In these models, missingness emerged as the second most important predictor for the dynamic feature model, and the most important predictor for the mean model (eFigure 4 and eFigure 5 in the Supplement).

## Discussion

There were 3 key findings in this study. First, using EMA data greatly improved prediction of posthospital suicide attempts beyond traditional approaches, and models including dynamic changes over time performed particularly well. Second, the strongest predictors were probability of acute change in suicidal thoughts or intentions. Third, missingness greatly increased predictive accuracy for both the mean and dynamic feature models. Each of these findings warrants further comment.

Model performance for the mean and dynamic feature models was substantially better than that for the static baseline model across all classification metrics. This suggests that collecting EMA data to obtain more temporal granularity in the measurement of suicidal thoughts can enhance our ability to predict suicide attempts. The dynamic feature model provided higher average AUC than the mean model, suggesting metrics of variability may be more important than central tendency. Indeed, variable importance metrics from the dynamic feature model indicated that the 3 most important predictors were probability of acute change in the desire to kill oneself, intent to kill oneself, and ability to resist the urge to kill oneself. Given that probability of acute change indexes the likelihood of an individual experiencing extreme shifts in suicidal thinking, rapid fluctuations in suicidal thinking may increase the risk of suicidal behaviors beyond average levels of suicidal thinking. This finding contrasts meta-analytic work^32^ from 2019 suggesting that metrics of variability do not meaningfully predict mental health symptoms beyond central tendency (ie, mean). There are several potential explanations for this discrepancy, including that this earlier work focused on affect dynamics, whereas we examined fluctuations in suicidal thinking. Furthermore, dynamic metrics may be more sensitive to acute perturbations (eg, psychiatric hospitalization).^33^ In addition, most previous work has focused on relatively healthy individuals, whereas participants in the current study were hospitalized for a recent suicide attempt or severe suicidal thoughts. It is possible that, in acutely suicidal patients, variability in suicidal thinking provides more information about suicide risk than average levels of suicidal thinking. This is supported by research on the importance of affective instability in other psychiatric populations (eg, eating disorders,^34^ borderline personality disorder^35^). Future research examining the benefits of temporally granular measurement and modeling dynamic features to predict suicide attempts could shed additional light on these important questions.

The dynamic feature model predicted posthospital suicide attempts with good accuracy (AUC, 0.89), which is at the high end of the range of reported AUC values for longitudinal prediction models of suicidal behaviors.^36^ Although machine learning methods have yielded high AUCs, the PPV of most models has been low. A 2019 review of 64 longitudinal suicide prediction models^36^ found most models reported extremely low (ie, <.01) PPVs, such that 99 out of every 100 individuals predicted by these models to attempt suicide will not. In comparison, the average cross-validated PPV for our dynamic feature model was 0.39. This represents a substantial improvement beyond both previous work and our baseline and mean models (although with still a long way to go) and was likely influenced by the relatively high base rate of suicide attempts in our sample. Prediction models with low PPVs can still have a positive net benefit if the benefits of interventions with true-positive results outweigh costs of intervening with false-positive results.^37^ Future studies with larger sample sizes are needed to replicate these results and determine whether models using EMA data can provide acceptable AUC and PPV scores to guide clinical decision-making and suicide prevention efforts.

As a secondary aim to explore the importance of missing EMA data, we added EMA missingness (ie, percentage compliance) as a predictor to both our mean and dynamic feature models, which greatly enhanced performance of both models (mean AUCs, 0.93). Notably, this missingness variable also emerged as the top predictor in the mean model and the second most important predictor in the dynamic feature model. These findings are consistent with prior work finding that nonresponse to questions about suicidal thinking is a particularly strong predictor of the transition from suicidal thinking to suicide attempts^14^ and highlight the importance of future studies taking an informative missingness approach for predicting suicidal behavior. However, caution is warranted in interpreting these findings, given that some evidence suggests using missingness for predictive modeling may introduce bias into models and generalize poorly to new data sets.^38^

### Limitations

These findings should be considered in the context of several limitations. First, we had a relatively small sample size (83 participants) that was predominantly White (83.1%). Future studies would benefit from larger sample sizes and longer follow-up periods, particularly given that suicide attempts are a low base-rate behavior, as well as more diverse and representative samples. Second, we only included self-reported measures of suicidal thoughts and urges; future research incorporating other self-reported measures (eg, affect) alongside psychophysiological and smartphone data (eg, heart monitor, accelerometer) may produce stronger models. Third, although the within-sample design of this study allowed for more than 1300 individual observations, we had a relatively small number of observations for some participants (median, 18.50 EMA responses; range, 3-120 EMA responses). This was partly due to the intentional naturalistic design of the study, with participants completing EMA prompts only for the duration of their hospital stay, which differed in length for participants. Nonetheless, overall compliance rate was relatively low (mean, 52.2%), and many missing responses may not have occurred at random. For instance, it is possible that participants did not complete responses when they were meeting with members of their care team (eg, therapist, psychiatrist) or were highly distressed. Finally, only 65 of the original 83 participants completed a follow-up survey, and we did not have access to claims and/or admissions data for all other hospitals in the area. Replicating these findings and models with more stringent compliance protocols^39^ could shed further light on the potential for effective risk detection in hospitals using EMA. Alongside future work on effective interventions and clinical guidelines, this could provide important information to identify and provide patients with support in a setting where care is already available and accessible.

## Conclusions

The current study provides novel insight on the high-risk period immediately following discharge from psychiatric hospitalization. These findings suggest that collecting dynamic, real-time data from patients during hospitalization can significantly improve the prediction of posthospitalization suicide attempts. Such information could be vital in clinical decision-making about discharge and posthospital level of care. Continued research incorporating temporally granular assessments of suicidal thoughts during high-risk time periods may advance the understanding, prediction, and prevention of suicide.

## Notes

Supplement. **eTable. **Model Coefficients for the Best Elastic Net Model Using Dynamic Ecological Momentary Assessment Features of Suicidal Thinking to Predict Posthospitalization Suicide Attempts **eFigure 1. **Individual Time-Series Plots of All Real-Time Suicidal Thoughts **eFigure 2. **Model Performance Metrics With 10-Fold Cross-Validation Repeated 3 Times **eFigure 3. **Model Performance Metrics for Leave-One-Out Cross-Validation **eFigure 4.** Feature Model Variable Importance for Mean-Only Model With Missingness **eFigure 5.** Feature Model Variable Importance for Dynamic Model With Missingness
