# Supplementary material for: A Pilot Study Using Frequent Inpatient Assessments of Suicidal Thinking to Predict Short-Term Postdischarge Suicidal Behavior
Source: JAMA Netw Open. 2021 Mar 9;4(3):e210591. doi: 10.1001/jamanetworkopen.2021.0591 (PMC7944382; doi:10.1001/jamanetworkopen.2021.0591)
Supplement: Supplement. — eTable. Model Coefficients for the Best Elastic Net Model Using Dynamic Ecological Momentary Assessment Features of Suicidal Thinking to Predict Posthospitalization Suicide Attempts eFigure 1. Individual Time-Series Plots of All Real-Time Suicidal Thoughts eFigure 2. Model Performance Metrics With 10-Fold Cross-Validation Repeated 3 Times eFigure 3. Model Performance Metrics for Leave-One-Out Cross-Validation eFigure 4. Feature Model Variable Importance for Mean-Only Model With Missingness eFigure 5. Feature Model Variable Importance for Dynamic Model With Missingness [file jamanetwopen-e210591-s001.pdf]

Supplemental Online Content

Wang SB, Coppersmith DDL, Kleiman EM, et al. A pilot study using frequent inpatient assessments of suicidal thinking to predict short-term postdischarge suicidal behavior. *JAMA Netw Open*. 2021;4(3):e210591. doi:10.1001/jamanetworkopen.2021.0591

eTable. Model Coefficients for the Best Elastic Net Model Using Dynamic Ecological Momentary Assessment Features of Suicidal Thinking to Predict Posthospitalization Suicide Attempts
eFigure 1. Individual Time-Series Plots of All Real-Time Suicidal Thoughts
eFigure 2. Model Performance Metrics With 10-Fold Cross-Validation Repeated 3 Times
eFigure 3. Model Performance Metrics for Leave-One-Out Cross-Validation
eFigure 4. Feature Model Variable Importance for Mean-Only Model With Missingness
eFigure 5. Feature Model Variable Importance for Dynamic Model With Missingness

This supplemental material has been provided by the authors to give readers additional information about their work.

© 2021 Wang SB et al. *JAMA Network Open.*

eTable. Model Coefficients for the Best Elastic Net Model Using Dynamic Ecological Momentary Assessment Features of Suicidal Thinking to Predict Posthospitalization Suicide Attempts

Predictor	Coefficient
(Intercept)	-6.75
min desire	0.57
mean desire	-0.18
max desire	–
SD desire	-1.70
MSSD desire	–
PUV desire	–
max change desire	0.30
PAC desire	24.67
min intent	-0.39
mean intent	-0.59
max intent	-0.01
SD intent	-0.17
MSSD intent	–
max change intent	–
PUV intent	0.07
PAC intent	2.61
min resist urge	–
mean resist urge	–
max resist urge	1.13
SD resist urge	0.48
MSSD resist urge	0.02
PUV resist urge	-0.03
max change resist urge	-0.06
PAC resist urge	2.92

Note. Tuning parameters for this best model were alpha = 0.65 and lambda = 0.01. SD = standard deviation; MSSD = mean square successive difference; PAC = probability of acute change; PUV = percent unique values; desire = desire to die by suicide ("How intense is your desire to kill yourself right now?"); intent = intent to die by suicide ("How strong is your intention to kill yourself right now?"); resist = ability to resist the urge to die by suicide ("How strong is your ability to resist the urge to kill yourself right now?").

© 2021 Wang SB et al. *JAMA Network Open.*

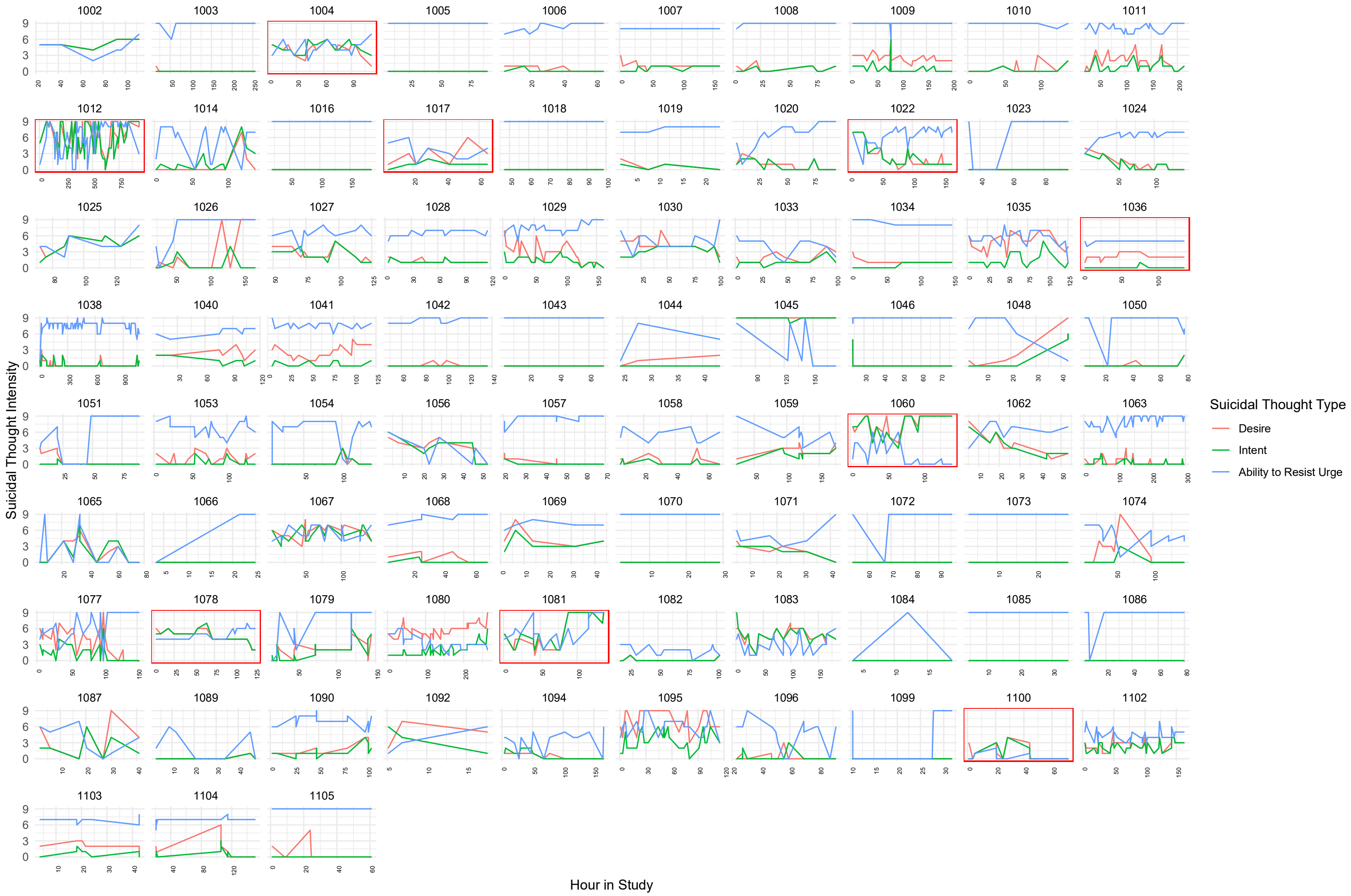

eFigure 1. Individual Time-Series Plots of All Real-Time Suicidal Thoughts
Red outlines indicate participants who attempted suicide in the four weeks following discharge from inpatient hospitalization.

© 2021 Wang SB et al. *JAMA Network Open.*

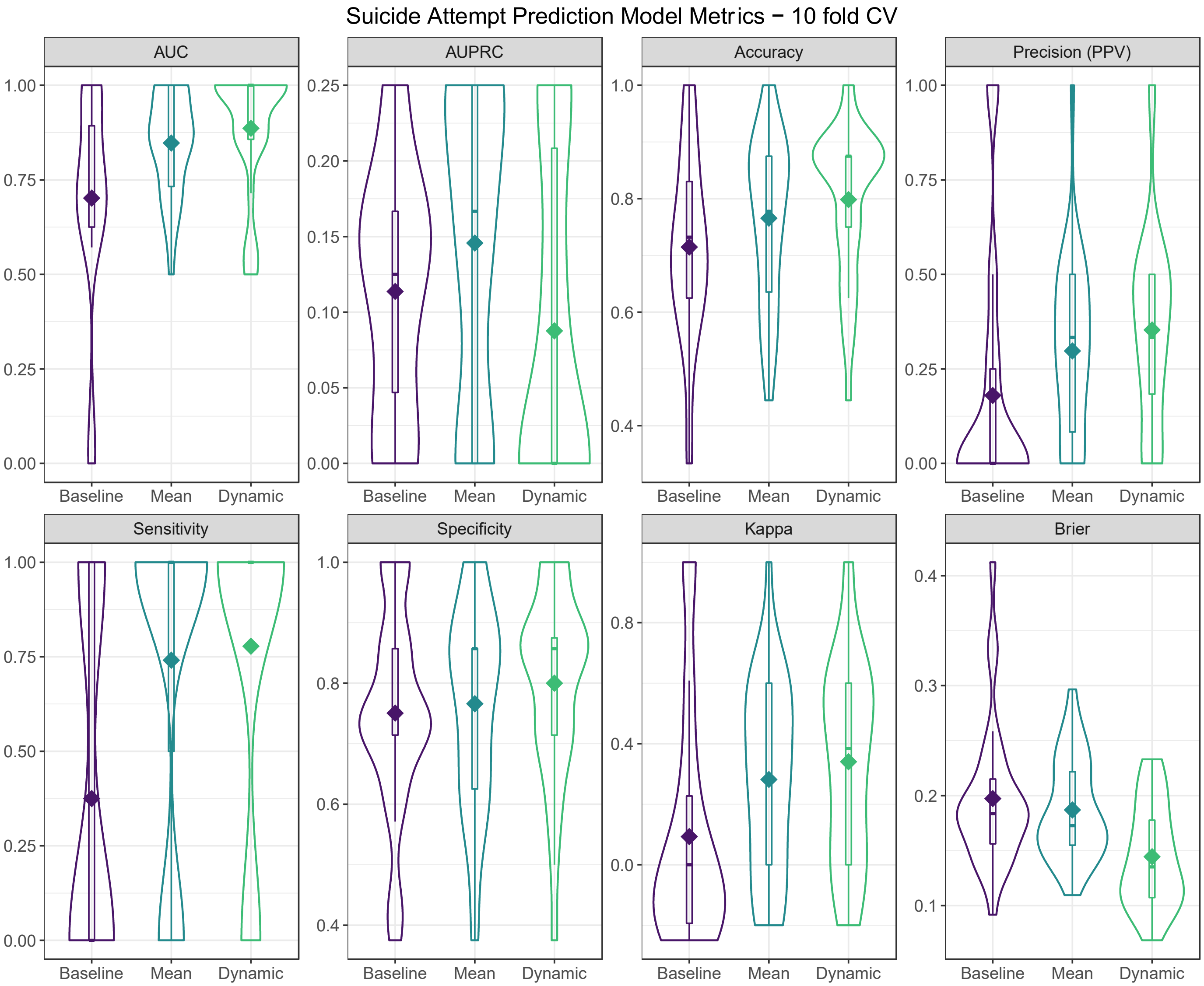

eFigure 2. Model Performance Metrics With 10-Fold Cross-Validation Repeated 3 Times
AUC = area under the receiver operating characteristic curve, AUPRC = area under the precision-recall curve.

© 2021 Wang SB et al. *JAMA Network Open.*

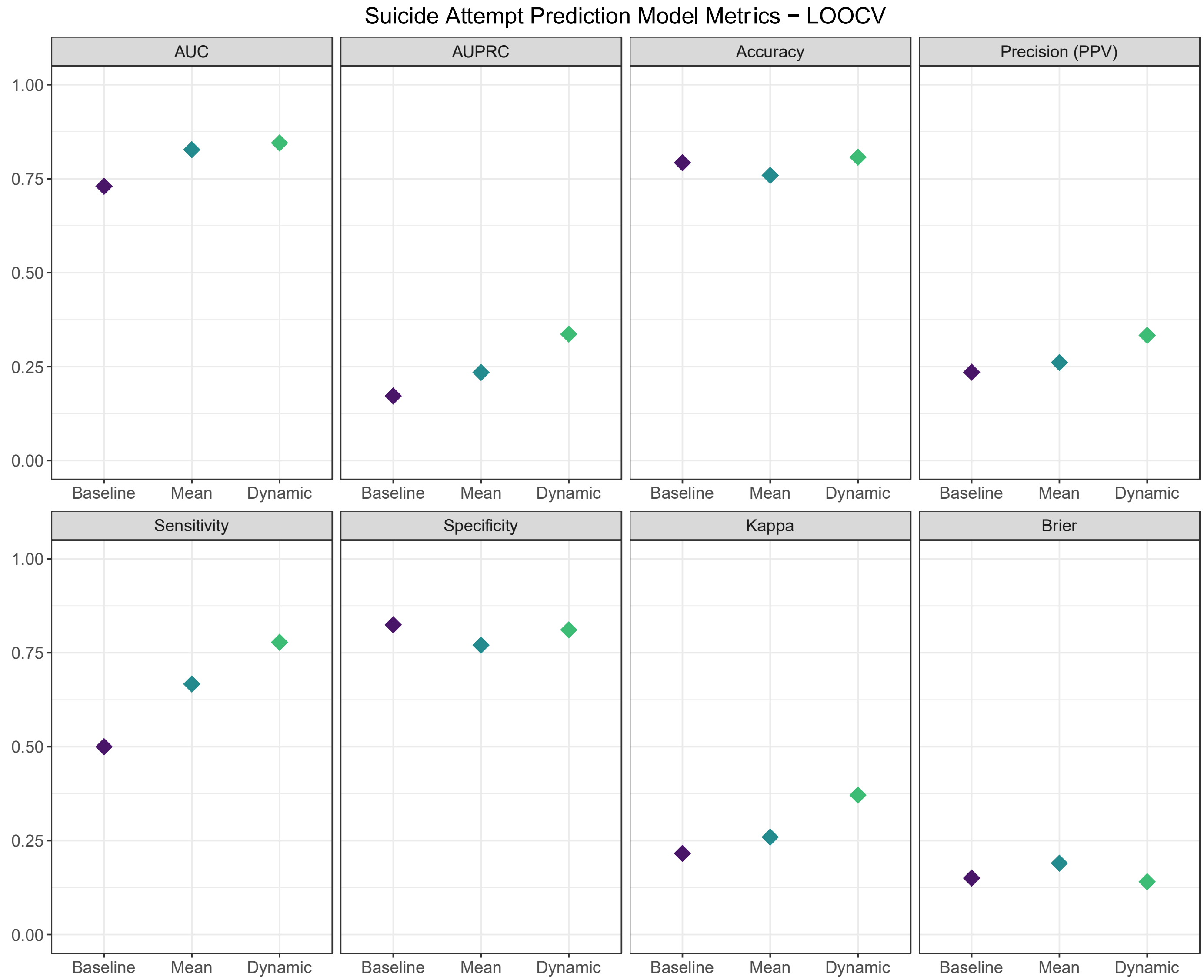

eFigure 3. Model Performance Metrics for Leave-One-Out Cross-Validation
AUC = area under the receiver operating characteristic curve, AUPRC = area under the precision-recall curve.

© 2021 Wang SB et al. *JAMA Network Open.*

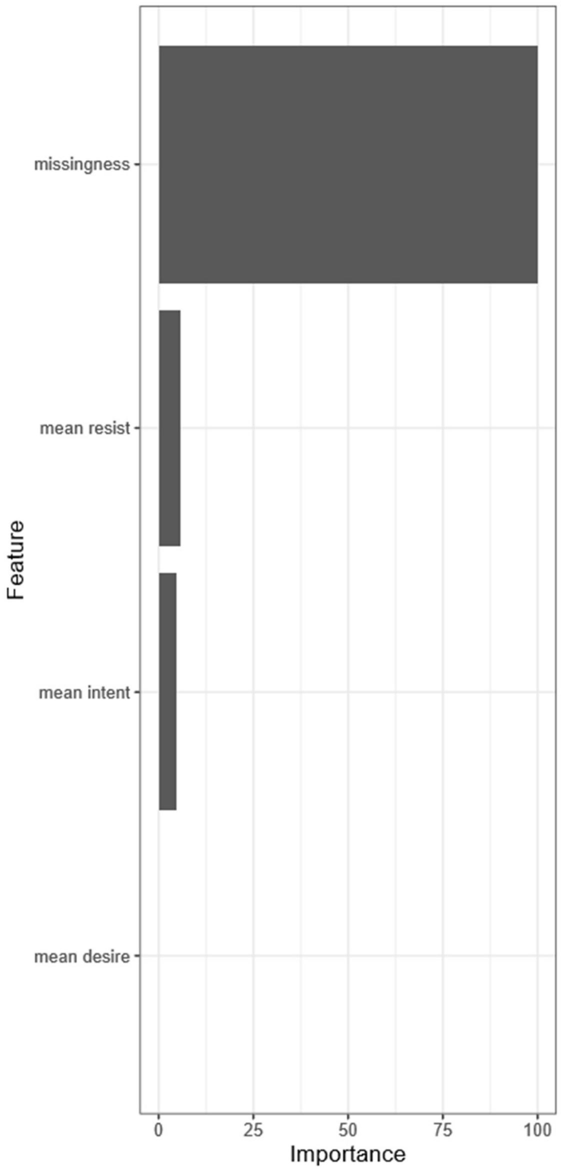

eFigure 4. Feature Model Variable Importance for Mean-Only Model With Missingness

© 2021 Wang SB et al. *JAMA Network Open.*

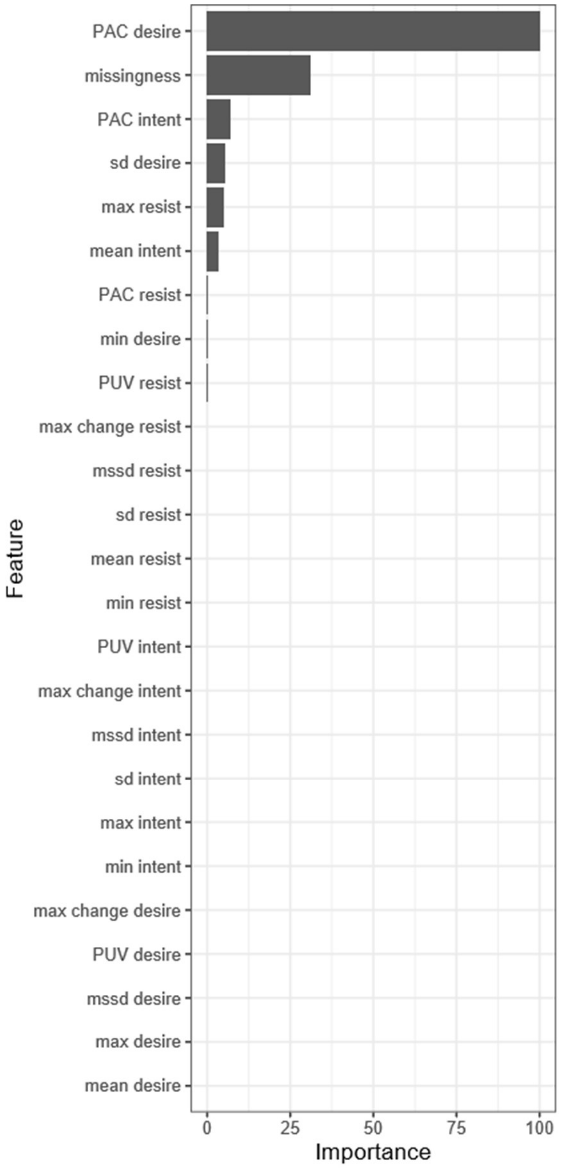

eFigure 5. Feature Model Variable Importance for Dynamic Model With Missingness

© 2021 Wang SB et al. *JAMA Network Open.*
